# PI3K/Akt pathway mediates the positive inotropic effects of insulin in Langendorff-perfused rat hearts

**DOI:** 10.1038/s41598-022-14092-2

**Published:** 2022-06-13

**Authors:** Yosuke Nakadate, Akiko Kawakami, Hiroaki Sato, Tamaki Sato, Takeshi Oguchi, Keisuke Omiya, Toru Matsuoka, Thomas Schricker, Takashi Matsukawa

**Affiliations:** 1 Department of Anesthesiology, University of Yamanashi, 1110 Shimokato, Chuo-City, Yamanashi 409-3898 Japan; 2 Department of Anesthesiology, University of Tsukuba Hospital, 2-1-1 Amakubo, Tsukuba-City, Ibaraki 305-8535 Japan; 3 Department of Anesthesia, McGill University Health Centre Glen Site, Royal Victoria Hospital 1001 Blvd, Decarie, Montreal, QC H4A 3J1 Canada

**Keywords:** Pharmacology, Cardiology, Endocrinology, Circulation

## Abstract

Insulin exerts positive inotropic effects on cardiac muscle; however, the relationship between cardiac contractility and phosphoinositol-3-kinase/Akt (PI3K/Akt) activation remains unclear. We hypothesized that the positive inotropic effects of insulin are dose-dependent and mediated via the PI3K/Akt pathway in isolated normal rat hearts. The Institutional Animal Investigation Committee approved the use of hearts excised from rats under pentobarbital anesthesia. The hearts were perfused at a constant pressure using the Langendorff technique. After stabilization (baseline), the hearts were randomly divided into the following four insulin (Ins) groups: 1) Ins0 (0 IU/L), 2) Ins0.5 (0.5 IU/L), 3) Ins5 (5 IU/L), and 4) Ins50 (50 IU/L) (n = 8 in each group). To clarify the role of the PI3K/Akt pathway in insulin-dependent inotropic effects, we also treated the insulin groups with the PI3K inhibitor wortmannin (InsW): 5) InsW0 (0 IU/L), 6) InsW0.5 (0.5 IU/L), 7) InsW5 (5 IU/L), and 8) InsW50 (50 IU/L). Hearts were perfused with Krebs–Henseleit buffer solution with or without wortmannin for 10 min, followed by 20 min perfusion with the solution containing each concentration of insulin. The data were recorded as the maximum left ventricular derivative of pressure development (LV dP/dt max). Myocardial p-Akt levels were measured at 3 min, 5 min, and at the end of the perfusion. In the Ins groups, LV dP/dt max in Ins5 and Ins50 increased by 14% and 48%, respectively, 3 min after insulin perfusion compared with the baseline. Tachyphylaxis was observed after 10 min in the Ins5 and Ins50 treatment groups. Wortmannin partially inhibited the positive inotropic effect of insulin; although insulin enhanced p-Akt levels at all time points compared with the control group, this increase was suppressed in the presence of wortmannin. The positive inotropic effect of insulin is dose-dependent and consistent with Akt activation. This effect mediated by high doses of insulin on cardiac tissue was temporary and caused tachyphylaxis, potentially triggered by Akt overactivation, which leads beta 1 deactivation.

## Introduction

Many inotropic agents, such as ephedrine, dopamine, and dobutamine^1^, exert dose-dependent, positive inotropic effects and are commonly used during surgery under general anesthesia^2^. Most of these agents stimulate beta receptors, leading to protein kinase A activation. However, they increase myocardial oxygen consumption, cause arrhythmia, and exert no cardioprotective effects. Therefore, the use of positive inotropic agents that exert their effects through other pathways would be better for anesthesia maintenance.

Insulin also exerts positive inotropic effects on cardiac muscle in animal models^3–5^. In addition, hemodynamic effects of high doses of insulin have been reported in healthy volunteers^6^. Insulin activates the phosphoinositol-3-kinase/Akt (PI3K/Akt) pathway and the substrates of Akt influence every aspect of cellular function, including growth, survival, proliferation, metabolism, glucose uptake, gene expression, and cell–cell communication by initiating the production of paracrine and autocrine factors^7^.

Insulin promotes utilization of glucose as the main cardiac energy substrate and can reduce coronary artery resistance and myocardial oxygen consumption, increase cardiac efficiency^8^, while Akt activates calcium handling^9^ and glucose uptake^8^. Therefore, the positive inotropic effect of insulin is believed to be mediated via the PI3K/Akt pathway. However, it is unknown whether cardiac contractility and Akt activation increase with insulin concentration. Therefore, we hypothesized that insulin could activate Akt in a dose-dependent manner to mediate its inotropic effects in the heart. In the present study, we assessed the relationship between insulin concentration and the inotropic effects mediated by the PI3K/Akt pathway in an isolated rat heart model, which allows testing the effect of different cardiovascular drugs on the coronary vasculature, muscle contraction, and heart rate (HR), without the interference of any other organ^10^.

## Methods

The experimental protocol used in this study was reviewed and approved by the University of Yamanashi Animal Care Committee (Protocol number A26-19). All methods were carried out in accordance with the guidelines of the “Animal experiment rules in University of Yamanashi” established by the Animal experimentation facility, University of Yamanashi. The study was carried out in compliance with the ARRIVE guidelines.

### Langendorff technique for isolated heart perfusion

Male Wistar rats (9 weeks old, 300–320 g) were anesthetized via intraperitoneal injection of 80 mg/kg pentobarbital sodium. The hearts were isolated and promptly immersed in cold modified Krebs–Henseleit (KH) buffer containing 118 mmol/L NaCl, 25 mmol/L NaHCO_3_, 4.7 mmol/L KCl, 1.2 mmol/L KH_2_PO_4_, 1.2 mmol/L MgSO_4_, 2.0 mmol/L CaCl_2_, 0.5 mmol/L di-NaEDTA, and 5.5 mmol/L glucose at 4 °C. The aorta was cannulated, and retrograde arterial perfusion was established at a constant pressure of 65 mm Hg with modified KH buffer. The KH solution was oxygenated with a mixture of 95% O_2_ and 5% CO_2_ and maintained at 37 °C. A small latex balloon was inserted into the left ventricle through the mitral valve and connected to a pressure transducer (TruWave, Edwards Lifesciences, CA, USA) to continuously monitor left ventricular (LV) pressure. The balloon volume was filled with water to maintain an LV end-diastolic pressure equivalent of 5–7 mmHg. A catheter was inserted into the pulmonary artery to collect the coronary venous return and measure the coronary flow.

### Experimental protocol

#### Experiment 1

**Figure 1:**
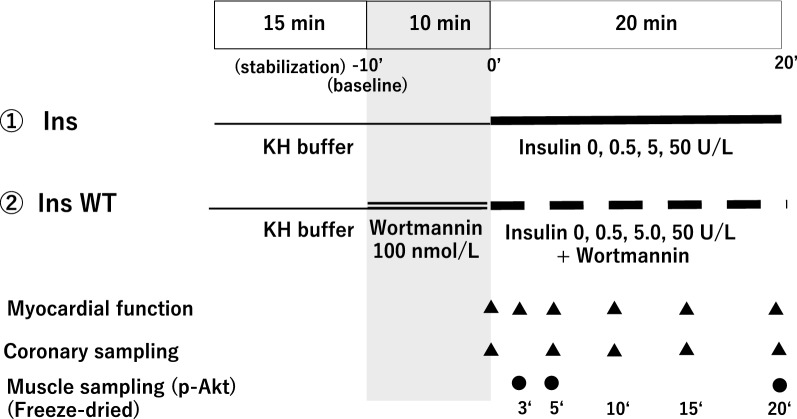
Experimental workflow for insulin perfusion in isolated rat hearts. In the insulin treatment (Ins) groups, hearts were perfused for 10 min with Krebs–Henseleit (KH) buffer containing 5.5 mmol/L glucose, followed by 20 min perfusion with each concentration of insulin (Experiment 1). In the insulin plus wortmannin treatment (InsW) groups, the hearts were perfused for 10 min with KH buffer containing 100 nmol/L wortmannin and 5.5 mmol/L glucose along with each concentration of insulin (Experiment 2). The hearts were then perfused for 20 min with a solution of each concentration of insulin and sampled for p-Akt measurement (Experiments 1 and 2). The HR and left ventricular derivative of pressure development (LV dP/dt) were measured 0, 3, 5, 10, 15, and 20 min after administration of each experimental perfusate, and the coronary effluent and partial pressure of oxygen were measured after 0, 5, 10, 15, and 20 min (Experiment 1 and 2). Whole heart muscles were sampled for p-Akt measurement at 3 and 5 min of insulin or control solution administration (Experiment 3).

To investigate the dose-dependent positive inotropic effect of insulin, the HR and contractility of the isolated rat hearts were allowed to stabilize for 15 min to achieve stable hemodynamics, after which the hearts were randomly assigned to the following four groups (n = 8 for each group): 1) Insulin 0 IU/L (Ins0; Control), 2) Insulin 0.5 IU/L (Ins0.5), 3) Insulin 5 IU/L (Ins5), and 4) Insulin 50 IU/L (Ins50). The exclusion criteria were HR < 200 bpm, a maximum LV pressure derivative (LV dP/dt max) < 2000 mmHg/s, and multiple arrhythmias after 10 min of stabilization. The experimental design is illustrated in Fig. 1. Hearts were perfused with KH buffer for 10 min, followed by perfusion with the insulin solutions for 20 min. Baseline hemodynamics were recorded just before perfusion, with each concentration started at 0 min. Following perfusion, the whole rat hearts were quickly frozen in liquid nitrogen and freeze-dried for 6 days before measuring p-Akt in the myocardial muscle.

#### Experiment 2

To clarify the role of the PI3K/Akt pathway in insulin-induced inotropic effects, we allocated hearts to the following four groups (n = 8 for each group) that were treated with wortmannin, a specific inhibitor of PI3Ks: 5) Insulin 0 IU/L (InsW0; Control), 6) Insulin 0.5 IU/L (InsW0.5), 7) Insulin 5 IU/L (InsW5), and 8) Insulin 50 IU/L (InsW50). In these four groups, the hearts were perfused with KH solution containing 100 nmol/L wortmannin for the first 10 min and then with the different insulin concentration solutions for 20 min. Following perfusion, the whole rat hearts were quickly frozen in liquid nitrogen and freeze-dried for 6 days.

#### Experiment 3

To measure the p-Akt value at 3 min and 5 min of insulin or control solution administration, whole heart muscles (n = 8 from each group) were sampled for p-Akt measurements using the technique described above (Fig. 1).

### Measurements

The HR (bpm) and LV dP/dt max (mmHg/s) were recorded continuously during the heart perfusions. Coronary flow (CF) was measured by timed collection of the perfusate from the catheter inserted into the pulmonary artery at baseline and immediately before (control) and after 5, 10, 15, and 20 min perfusion with insulin. Oxygen tension was measured using a blood gas analyzer (iSTAT1 analyzer; Abbott, JAPAN, Tokyo). Oxygen consumption was calculated using the formula described by Neely et al.^11^. Oxygen consumption (mmol/h per g) = ((arterial – venous oxygen tension) (mm Hg) / 760 (mm Hg)) × solubility of oxygen at 37 °C (ml/ml H_2_O) / 22.4 [ml/mmol]) × (CF [ml/h] / dry weight of heart [g]).

The freeze-dried myocardium was suspended in an assay lysis buffer (Lysis Buffer 6, R&D Systems, Minneapolis, MN, USA) containing phenylmethanesulfonyl fluoride (2 mM, Sigma-Aldrich, Inc., St. Louis, MO, USA) and a protease inhibitor cocktail (Sigma-Aldrich, Inc.). The samples were homogenized using a micro-homogenizing system (MicroSmash MS-100R, TOMY SEIKO Co., Ltd., Tokyo, Japan), and the homogenates were centrifuged for 5 min at 2000 × *g*^12^. The supernatants were assayed for p-Akt using enzyme-linked immunosorbent assay ([pSer^473/474^] Akt1/2, Enzo, Farmingdale, NY, USA). The concentrations of p-Akt were quantified spectrophotometrically (Spectra Max 340, Molecular Devices, Sunnydale, CA, USA) at 450 nm. The wavelength correction was set at 540 nm. The values are expressed as milligrams p-Akt per gram dry heart weight.

### Statistical analysis

Data are presented as mean ± standard deviation (SD). Intergroup comparisons for baseline measurements such as hemodynamic data and dry heart weight were performed using one-way analysis of variance (ANOVA). Changes in hemodynamics and the concentrations of p-Akt were analyzed using repeated two-way ANOVA with post-hoc Bonferroni test. Two-sided *P* values < 0.05 were considered statistically significant.

Sample size calculation was based on the expected difference in contractility (LV dP/dt max) between the groups. The results of a preliminary study showed a percent change in LV dP/dt max that was equivalent to 0% in the control group, 10% in the Ins0.5 group, 30% in the Ins5 group, and 70% in the Ins50 group 3 min after insulin administration. At least eight rats were required in each group to achieve a power level of 80%, with an alpha error of 5% (SD = 40%). All statistical analyses were performed using SPSS ver.23 for Windows (IBM, Chicago, IL, USA).

### Ethics approval and consent to participate

The study was approved by the Ethics Committee on Animal Research of the University of Yamanashi (Protocol number A26-19, December 22, 2014). Consent to participate is not applicable.

## Results

Hemodynamic data are presented in the figures. Intragroup differences are indicated using asterisks or dagger marks, and marks of intragroup differences have been omitted.

### Experiment 1: Effect of insulin dosage on hemodynamic parameters in the isolated rat heart

**Figure 2:**
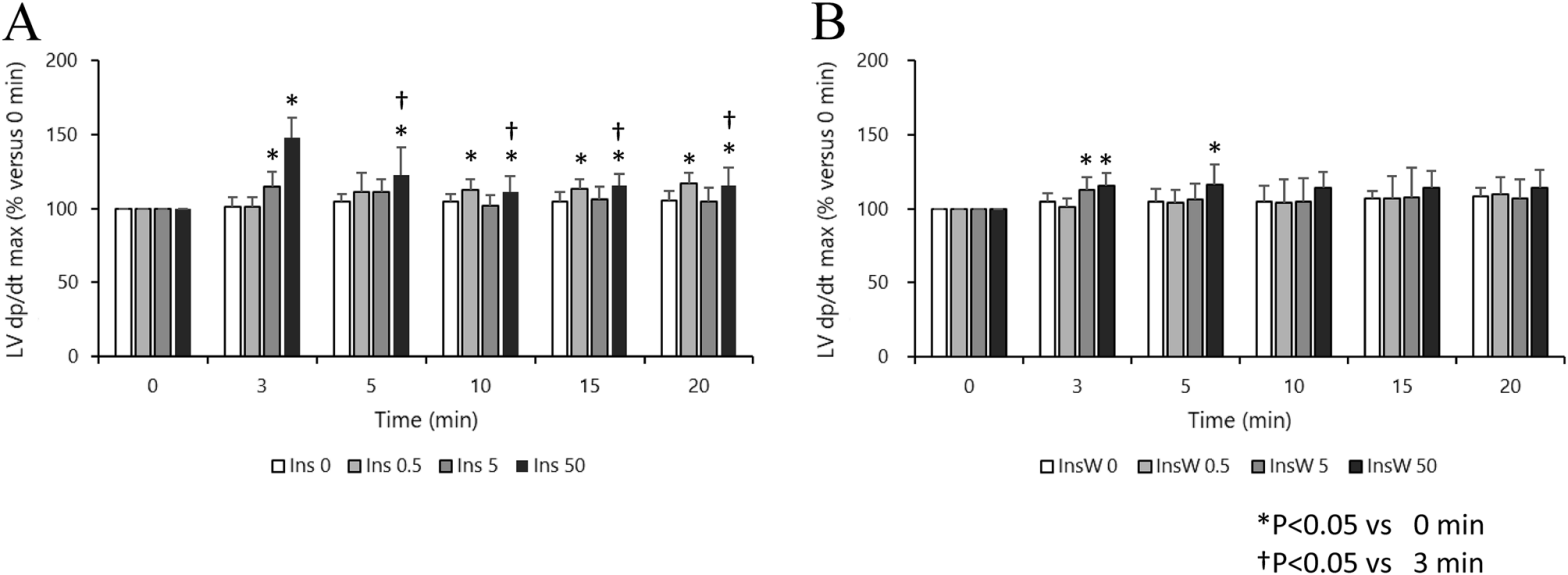
Effect of Insulin with or without wortmannin on the changes in LV dP/dt max over time in each group (n = 8). Insulin treatment groups (Ins) are shown in A. Insulin plus wortmannin treatment groups (InsW) are shown in B. LV dP/dt max for 5 and 50 IU/L insulin at 3 min and that for 50 IU/L at 5 min increased significantly compared to that in Ins 0 (control group; intergroup comparison) (**A**). LV dP/dt in Ins0.5 increased at 10 min and thereafter, that in Ins 5 increased at 3 min only, and that in Ins50 increased at all time points but those at 5–20 min were lower than those at 3 min (in the intragroup comparison) (**A**). In the presence of wortmannin, LV dP/dt in InsW50 was higher than that in InsW0, and those in InsW5 and Ins50 were higher than those in InsW0.5 at 3 min (**B**). LV dP/dt in InsW5 (at 3 min) and InsW50 (at 3 and 5 min) increased temporarily (in the intragroup comparison) (**B**) Data are presented as the mean ± SD. *P < 0.05 versus 0 min. ^†^*P* < 0.05 versus 3 min. Marks of intergroup differences have been omitted. LV dP/dt max (mmHg/s), maximum of left ventricular derivative of pressure development.

**Figure 3:**
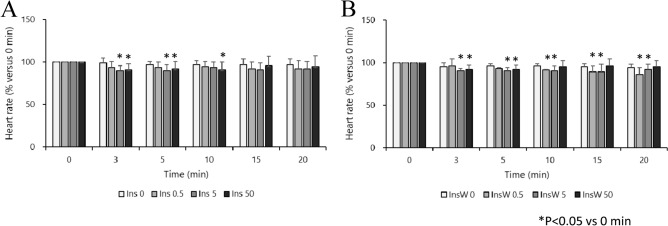
Effect of insulin with or without wortmannin on changes in the heart rate over time in each group (n = 8). Insulin treatment groups (Ins) are shown in (**A**). Insulin plus wortmannin treatment groups (InsW) are shown in B. There were no significant intergroup differences (**A**, **B**). In Ins 5 (3 and 5 min) and Ins50 (3, 5, and 10 min), HR was temporarily decreased (in the intragroup comparison) (**A**). HR in InsW0.5 was decreased at 10 min and thereafter, that in InsW5 was decreased at all time points, and that in Ins W50 was decreased within 10 min (in the intragroup comparison) (**B**). Data are presented as mean ± SD. *P < 0.05 versus 0 min.

**Figure 4:**
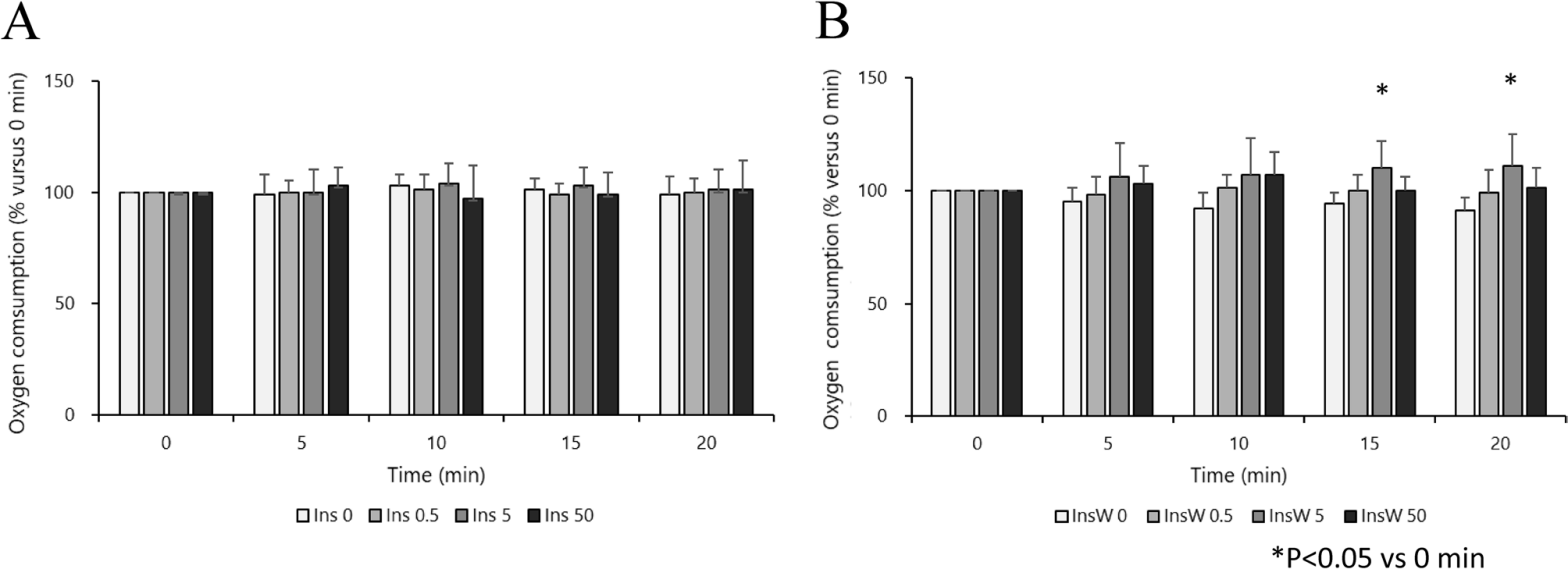
Effect of insulin with or without wortmannin on changes in oxygen consumption over time in each group (n = 8). Insulin treatment groups (Ins) are shown in A. Insulin plus wortmannin treatment groups (InsW) are shown in B. There were no significant intragroup and intergroup differences in oxygen consumption in the insulin group (**A**). There was no significant intergroup difference at any time point in the InsW groups. Oxygen consumption in InsWT5 increased at 15 and 20 min compared to that baseline, but this change was not observed in InsWT50 (in the intergroup comparison) (**B**). Data are presented as the mean ± SD.

There were no significant differences in baseline HR, LV dP/dt max, CF, and dry heart weight in each group (Table 1). High doses of insulin (5 and 50 IU/L) had a significant inotropic effect in the early phase (at 3 min), but tachyphylaxis was observed thereafter. The low dose of insulin (0.5 IU/L) had a weak but continuous inotropic effect (Fig. 2A). There were no significant intergroup differences in the HR. In the Ins5 (3 and 5 min) and Ins50 (3, 5, and 10 min) groups, HR decreased temporarily (Fig. 3A) compared with that at 0 min. The CF did not change significantly throughout the experiment in any of the treatment groups (Additional File 1A), neither did oxygen consumption in any of the insulin treatment groups (Fig. 4A).

**Table 1:** Dry heart weight and baseline measurements of Langendorff-perfused rat hearts.

	Ins0	Ins0.5	Ins5	Ins50
Number (n)	8	8	8	8
Dry heart weight (g)	0.23 ± 0.02	0.21 ± 0.03	0.25 ± 0.04	0.24 ± 0.02
HR (bpm)	258 ± 19	233 ± 30	247 ± 28	248 ± 33
LV dP/dt max (mmHg/sec)	2119 ± 291	1950 ± 236	2131 ± 200	2175 ± 219
CF (ml/min)	14.0 ± 1.4	13.1 ± 3.9	14.7 ± 2.3	14.6 ± 2.8

	InsW0	InsW0.5	InsW5	InsW50
Number (n)	8	8	8	8
Dry heart weight (g)	0.21 ± 0.04	0.24 ± 0.05	0.25 ± 0.03	0.21 ± 0.05
HR (bpm)	263 ± 35	242 ± 32	249 ± 33	254 ± 25
LV dP/dt max (mmHg/sec)	2069 ± 173	2175 ± 189	2069 ± 248	2118 ± 371
CF (ml/min)	13.8 ± 2.7	13.4 ± 3.3	13.6 ± 2.8	12.7 ± 1.7

Data represent the mean ± SD. Baseline measurements are presented in absolute values obtained 10 min after stabilization, except for dry heart weight, which was measured at the end of the perfusion.

Ins, insulin treatment group; InsW, insulin and wortmannin co-treatment group; HR, heart rate; LV dP/dt max, maximum left ventricular derivative of pressure development; CF, coronary flow.

Therefore, insulin had a dose-dependent positive inotropic effect. The effect of low-dose insulin was continuous, while that of high-dose insulin was transient, and the HR was also temporarily decreased. The inotropic effect was exerted without increasing oxygen consumption.

### Experiment 2: Roles of pAkt activation on inotropic effect in isolated rat heart

In the presence of wortmannin, there were no significant differences in baseline HR, LV dP/dt max, CF and dry heart weight in each group (Table 1). High doses of insulin (InsW 5 and InsW50) showed a temporary inotropic effect (at 3 min and at 3 and 5 min, respectively) even in the presence of wortmannin; however, the inotropic effect mediated by low doses of insulin was inhibited (Fig. 2B). The HR decreased after 10, 15, and 20 min in InsW0.5, at all time points in InsW5, and after 3 and 5 min in InsW50 (Fig. 3B). The CF did not change significantly throughout the experiment in any of the wortmannin treatment groups (Additional File 1B). Oxygen consumption in InsW5, but not in InsW50, increased by only 10% and 11% at 15 and 20 min, respectively, compared to that at baseline (Fig. 4B). Hence, wortmannin, a PI3K specific inhibitor, partially inhibited the positive inotropic effect of insulin.

### Experiment 3: Insulin concentration with and without PI3K inhibitor and Akt activation at different time points

**Figure 5:**
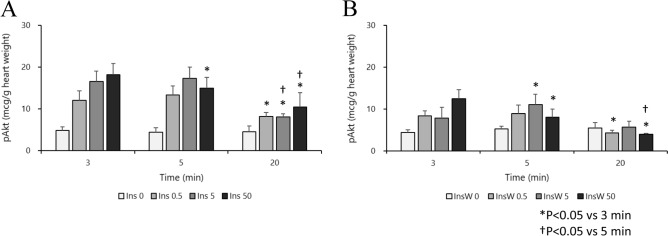
Effect of insulin with or without wortmannin on changes in p-Akt levels over time in each group (n = 8). Insulin treatment groups (Ins) are shown in A. Insulin plus wortmannin treatment groups (InsW) are shown in B. p-Akt levels were increased at all time points compared with those in the control group (in the intergroup comparison) (**A**). In all insulin groups, p-Akt levels at the later time point (at 20 min) were decreased compared to those at 3 min (in the intragroup comparison) (**A**); in the presence of wortmannin, this change was only noticed in at the early time points (3 and 5 min) (in the intergroup comparison) (**B**). In all insulin groups in the presence of wortmannin, pAkt levels at the later time points (at 5 or 20 min) were also decreased compared to those at 3 min (in the intragroup comparison) (**B**). Data are presented as the mean ± SD. Marks of intergroup differences have been omitted *P < 0.05 versus 3 min. ^†^*P* < 0.05 versus 5 min.

In all insulin groups, p-Akt levels were increased at all time points compared with that in the control group (Fig. 5A). However, in all insulin groups, p-Akt levels in the later time points (at 20 min) were decreased compared to those at 3 min (Fig. 5A). In the presence of wortmannin, this increase was observed only in the early phases (3 and 5 min) of these experiments (Fig. 5B). In all insulin groups, pAkt levels in the presence of wortmannin in the later time points (at 5 or 20 min) were also decreased compared to those at 3 min (Fig. 5A).

Akt phosphorylation and cardiac contractility increased concomitantly with insulin concentration during the early phase of treatment, but some discrepancies were observed between the p-Akt level and myocardial performance during the late phase (at 5 and 20 min).

## Discussion

In this study, on assessing the relationship between insulin concentration and the inotropic effects mediated by the PI3K/Akt pathway in an isolated rat heart model, we found that the positive inotropic effect of insulin during the early time points (within 3 min) was dose-dependent and was likely mediated by the PI3K/Akt pathway because of a concomitant increase in Akt phosphorylation and cardiac contractility. In contrast, tachyphylaxis was detected in groups with high doses of insulin (Ins5 and Ins50). Low doses of insulin (Ins0.5 group) exerted weak but continuous positive inotropic effects even in the later time points (at 5 and 20 min). These findings suggest that factors other than pAkt are involved in the mechanism of tachyphylaxis because some discrepancies were observed between p-Akt levels and myocardial performance.

Inotropy is modulated by calcium following its entry into myocardial cells via L-type calcium channels, calcium release by the sarcoplasmic reticulum (SR) via the activation of ryanodine receptors (RyR), calcium binding to protein, troponin C, myosin phosphorylation, SR Ca^2+^ (SERCA) ATPase activity, and calcium efflux across the sarcolemma mediated by an ATP-dependent Ca^2+^ pump Na^+^/Ca^2+^ exchanger and Na/K/ATPase pump^13^. Insulin increases calcium entry through L-type channels^14^ and Na^+^/Ca^2+^ exchangers^15^. In addition, insulin increases the mRNA expression of RyR and SERCA ATPase^16^. These improvements in calcium binding and/or trans-sarcolemma calcium transportation are mediated by PI3K/Akt activation. Insulin binding to its receptor leads to the activation of the PI3K/Akt pathway, which is a main player in mediating the metabolic effects of insulin. Several reports have shown that Akt phosphorylation also regulates L-type calcium channels^17^, RyR activation^18^, myosin phosphorylation^9^, and (SERCA) ATPase activity^19^. When a calcium in perfusate was used within a biological range (1.25 mmol/L), the inotropic effects of insulin were mediated dose dependently in isolated rat hearts^20^. Our results demonstrated that Akt activation was almost consistent with cardiac contractility 3 min after insulin administration, thereby contributing to enhanced inotropic effects.

Indeed, the inotropic effect of low dose insulin (0.5 IU/L) was not observed in the presence of a specific inhibitor of PI3Ks, further supporting the role of Akt activation in inotropic effects in the normal rat heart.

However, the positive inotropic effects were temporary at high insulin concentrations, and the p-Akt values did not correlate with myocardial performance during the late phase of insulin treatment. Although p-Akt levels in the insulin groups were higher than those in the control group at 20 min, cardiac contractility and oxygen consumption were comparable among all groups at 20 min. A previous study reported that the inotropic effect under a supraphysiological insulin concentration (25 IU/L) was abolished when the heart was perfused with insulin for 10 min^21^. It was speculated that glycolytic ATP formation plays a key role in the development of insulin-induced tachyphylaxis. The heart requires a constant supply of fuel and oxygen to maintain its intracellular ATP level and the myocardial contraction/relaxation cycle^22^. In our study, oxygen and glucose was supplied continuously by oxygenated perfusate and oxygen consumption, which was shown in additional file 1, was similar independent of insulin concentration or presence of wortmannin. Our data also revealed a higher pAkt level at all time points in the insulin groups than in the control group. It suggested that ATP production should increase in the presence of insulin because glucose uptake through glucose transporters (GLUTs; mainly GLUT4 in adult heart) is promoted via PI3K/Akt activation in a dose-dependent manner^8,22^ when glucose and insulin concentrations increase. Therefore, other factors are likely to influence high-dose insulin-induced tachyphylaxis, such as cAMP. Recently. Oudit et al. reported that the loss of PI3K can increase basal myocardial cAMP levels and myocardial expression of adenylate cyclase^23^. Moreover, PI3K signaling negatively regulates β-adrenergic positive inotropic effects^24^; therefore, insulin-induced tachyphylaxis may be observed when cAMP suppression following Akt activation supersedes calcium handling activation. This hypothesis may explain the partial suppression of Akt activation, ameliorated inotropic effect of insulin, and lack of tachyphylaxis in hearts pre-treated with wortmannin. However, further studies are needed to clarify the occurrence of tachyphylaxis.

We observed decreased HR in the presence of high doses of insulin. The HR depends on the rate at which the sinoatrial node (SA) produces action potentials, while β1 adrenoceptors, M2 muscarinic receptors, and messengers play important roles in regulating it. Activation of β1 adrenoceptors expressed on the membrane of pacemaker cells activate Gs-proteins, leading to increased levels of cAMP, which binds to hyperpolarization-activated cyclic nucleotide-gated channels that increase the current and rate of depolarization during pacemaker potential. cAMP also increases the Ca^2+^ current, opening time of L-type calcium channels, and the speed of phase 0. Acetylcholine binds to the M2 muscarinic receptor and activates the coupled Gi-protein, which increases K^+^ flow out of the cell and decreases the membrane potential, increasing the time taken by pacemaker cells to reach their threshold values^25^. Lin et al. reported that PI3K activation increases the HR independent of the autonomic nervous system because Akt inhibition slows the pacing rate of the SA^26^. Conversely, Akt also stimulates the muscarine receptor^18^ and Gi-proteins^27^, which suggests that the muscarinic effect may be dominant in high doses of insulin compared to increased current.

In this study, insulin enhanced myocardial contractility without increasing myocardial oxygen consumption. We have previously reported that insulin exerts an ischemic protective effect on the same isolated rat heart model^12^ and, therefore, an inotropic effect mediated by the PI3K/Akt pathway might maintain hemodynamics during general anesthesia. We need further study such as effects of combination of a lower dose of insulin with catecholamines and /or for female rats for clinical application.

This study had some limitations. First, the insulin 5 and 50 IU/L concentrations were supraphysiologic. Second, the dose of wortmannin used might not have been sufficient to completely inhibit Akt activation. Third, we did not measure the downstream effects of the PI3K/Akt pathway and, thus, further studies are required to clarify the details underlying its downstream effects. Fourth, although in our model we constantly supplied 5.5 mmol/L of glucose, we did not consider the risk of hypoglycemia. In the clinical setting, meticulous glucose administration would be required to avoid hypoglycemia.

## Conclusion

Insulin activates the PI3K/Akt pathway in a dose-dependent manner, which leads to increased cardiac contractility in isolated intact rat hearts. High doses of insulin exerted transient inotropic effects, which could be reduced by wortmannin treatment. Further studies are needed to clarify the downstream events of the tachyphylaxis pathway.

## Supplementary Information

Supplementary Information 1.

Supplementary Information 2.

## Data Availability

The datasets used and analyzed during this study are available from the corresponding author upon reasonable request.

## Abbreviations

ANOVA: Analysis of variance
GLUT: Glucose transporter
HR: Heart rate
KH: Krebs–Henseleit
LV: Left ventricular
LV dP/dt max: Maximum LV pressure derivative
Ins0: Insulin 0 IU/L
Ins0.5: Insulin 0.5 IU/L
Ins5: Insulin 5 IU/L
Ins50: Insulin 50 IU/L
InsW0: Insulin 0 IU/L and wortmannin
InsW0.5: Insulin 0.5 IU/L and wortmannin
InsW5: Insulin 5 IU/L and wortmannin
InsW50: Insulin 50 IU/L and wortmannin
PI3K/Akt: Phosphoinositol-3-kinase/Akt
RyR: Ryanodine receptors
SA: Sinoatrial node
SD: Standard deviation
SERCA: Sarcoplasmic reticulum Ca^2+^
SR: Sarcoplasmic reticulum
