# Supplementary material for: PI3K/Akt pathway mediates the positive inotropic effects of insulin in Langendorff-perfused rat hearts
Source: Sci Rep. 2022 Jun 13;12:9793. doi: 10.1038/s41598-022-14092-2 (PMC9192604; doi:10.1038/s41598-022-14092-2)
Supplement: Supplementary file 1 — Supplementary Information 1. [file 41598_2022_14092_MOESM1_ESM.docx]

**Additional File 1 Effect of insulin with or without wortmannin on changes in coronary flow over time in each group** (n = 8). Insulin treatment groups (Ins) are shown in A. Insulin plus wortmannin treatment groups (InsW) are shown in B. In all groups, there were no significant intergroup and intragroup differences (A,B). Data are presented as mean ± SD.
