# Supplementary figures and images for: PI3K/Akt pathway mediates the positive inotropic effects of insulin in Langendorff-perfused rat hearts
Source: Sci Rep. 2022 Jun 13;12:9793. doi: 10.1038/s41598-022-14092-2 (PMC9192604; doi:10.1038/s41598-022-14092-2)

## Slide 1
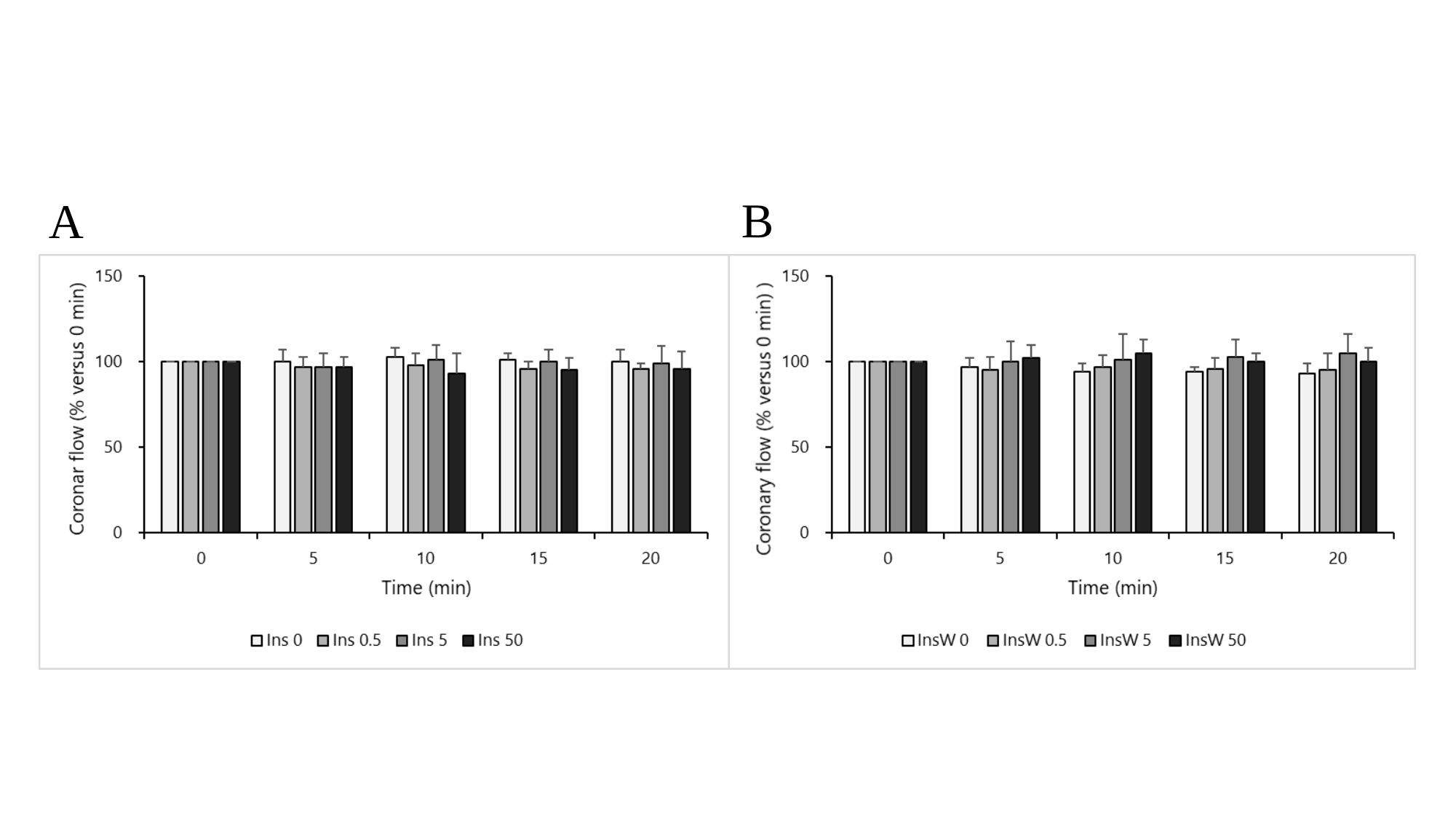

B
A

Supplement: Supplementary file 2 — Supplementary Information 2. [file 41598_2022_14092_MOESM2_ESM.pptx]
